# Amphibian (*Xenopus laevis*) Tadpoles and Adult Frogs Differ in Their Use of Expanded Repertoires of Type I and Type III Interferon Cytokines

**DOI:** 10.3390/v10070372

**Published:** 2018-07-17

**Authors:** Emily S. Wendel, Amulya Yaparla, Mattie L. S. Melnyk, Daphne V. Koubourli, Leon Grayfer

**Affiliations:** 1 Department of Biological Sciences, George Washington University, 800 22nd St NW, Suite 6000, Washington, DC 20052, USA; emilywendel@gwu.edu (E.S.W.); ayap@gwu.edu (A.Y.); daphnevk@gwu.edu (D.V.K.); 2 School Without Walls High School, Washington, DC 20052, USA; mattie.melnyk@gmail.com

**Keywords:** amphibian, interferon, IFN, FV3, antiviral

## Abstract

While amphibians around the globe are facing catastrophic declines, in part because of infections with pathogens such as the Frog Virus 3 (FV3) ranavirus; the mechanisms governing amphibian susceptibility and resistance to such pathogens remain poorly understood. The type I and type III interferon (IFN) cytokines represent a cornerstone of vertebrate antiviral immunity, while our recent work indicates that tadpoles and adult frogs of the amphibian *Xenopus laevis* may differ in their IFN responses to FV3. In this respect, it is notable that anuran (frogs and toads) tadpoles are significantly more susceptible to FV3 than adult frogs, and thus, gaining greater insight into the differences in the tadpole and adult frog antiviral immunity would be invaluable. Accordingly, we examined the FV3-elicited expression of a panel of type I and type III IFN genes in the skin (site of FV3 infection) and kidney (principal FV3 target) tissues and isolated cells of *X. laevis* tadpoles and adult frogs. We also examined the consequence of tadpole and adult frog skin and kidney cell stimulation with hallmark pathogen-associated molecular patterns (PAMPs) on the IFN responses of these cells. Together, our findings indicate that tadpoles and adult frogs mount drastically distinct IFN responses to FV3 as well as to viral and non-viral PAMPs, while these expression differences do not appear to be the result of a distinct pattern recognition receptor expression by tadpoles and adults.

## 1. Introduction

Escalating infections of amphibians by ranaviruses such as Frog Virus 3 (FV3; ranavirus genus and family *Iridoviridae*) are significantly contributing to the worldwide amphibian declines [1,2,3]. It is notable that the tadpoles of anurans (frogs and toads) are generally much more susceptible to ranaviruses than the adult frogs of the respective species [4,5,6,7,8]. Unfortunately, the immunological reasons behind these susceptibility differences, and in particular, the differences between anuran tadpole and adult frog antiviral immunity remain to be adequately understood.

Notably, the interferon (IFN) cytokines are very important to the antiviral immune responses of all vertebrates [9]. More recently diverged animal species such as reptiles, birds and mammals possess three types of IFN genes; type I, II, and III *IFNs* [10], of which the type I and type III IFNs are thought to be more exclusively involved in antiviral immunity [9]. While the type I IFNs of these animals are encoded by intronless transcripts, their type III IFNs (also known as IFNλs/IFNLs) are encoded by five exon/four intron pre-mRNAs [9]. By contrast, bony fish appear not to possess type III *IFNs*, while their type I *IFNs* exhibit the five exon/four intron organization adopted by reptilian, avian and mammalian type III *IFNs* [9]. Amphibians represent key stages in the evolution of these IFN cytokine families as they possess both type I *IFNs* with the five exon/four intron trancript organization seen in fish, as well as mammalian-like intron-containing type III *IFNs* [11]. Moreover, amphibians have recently been discovered to also possess intron-containing as well as intronless type I *IFNs* [12,13]. Indeed, as amphibians are phylogenetic intermediates between fish and mammals and possess fish-like and mammalian-like type I *IFNs*, as well as mammalian-like and unique type III *IFN* genes [12,13]; these animals offer a unique perspective into the evolution of vertebrate antiviral immunity [14,15,16]. In turn, greater insights into the respective roles of these highly expanded amphibian IFN cytokine families in tadpoles and adult anurans will undoubtedly lend to greater insight into tadpole susceptibility and adult frog resistance to amphibian pathogens like FV3. In this respect, it is notable that our recent work indicates that while tadpoles of the anuran *X. laevis* respond to FV3 infections by upregulating a type III *IFN X. laevis*, adults instead respond by upregulating a type I *IFN* [17,18,19]. 

Here, we expand on our previous work by examining the roles of a panel of intronless and intron containing type I and type III *IFNs* in the *X. laevis* tadpole and adult frog responses to FV3 challenge, as well as to a range of pathogen pattern recognition receptor agonists such as lipoprotein, lipopolysaccharide, polyinosinic:polycytidylic acid, CpG DNA, imiquimod, single stranded RNA and several viral DNAs.

## 2. Materials and Methods

### 2.1. Animals, Culture Media, and Conditions

Outbred tadpole and adult *Xenopus laevis* were purchased from the Xenopus 1 facility, and housed and handled under strict laboratory and IACUC regulations (Approval number 15-024, 30 September 2015).

The cell culture media and conditions have been previously described [20].

### 2.2. FV3 Stocks and Infections

The FV3 production has been described previously [21]. In brief, baby hamster kidney (BHK-21) cells were infected with FV3 (multiplicity of infection; MOI: 0.1), and grown at 30 °C and 5% CO_2_ for 5 days. The FV3-containing supernatants were collected over 30% sucrose by ultracetrifugation, re-suspended in saline, and the viral titers were determined by plaque assay over BHK-21 cells.

Tadpoles and the adult frogs were infected by water-bath containing 1 × 10^6^ PFU/mL of FV3 for 1 h in 100 mL of water, were washed in FV3-free water and housed for an additional 6 h. After 6 h, the animals were sacrificed by tricaine mesylate (TMS) overdose and the tissues were collected for RNA and DNA isolation. Mock-infections were carried out in parallel using cohort animals by exposing tadpoles and adults to equal volumes of FV3-free water for the respective amounts of time as above. 

### 2.3. Isolation of X. laevis Tadpole and Adult Frog Skin and Kidney Cells

The tadpole and adult frog skin and kidney tissue were excised and incubated in 7.5 μg/mL of liberase (Sigma, St. Louis, MO, USA) for 30 min at 27 °C. After this incubation, the samples were homogenized by passage through the progressively larger gage needles, washed with saline and the cells collected by centrifugation. The cells were then resuspended in a medium and enumerated. The cells were cultured in an amphibian medium Iscove’s Modified Dulbecco’s Medium containing Penicillin and Streptomycin (200 U/mL), gentamycin (15 μg/mL), non-essential amino acids, primatone, and 10% fetal bovine serum, and were adjusted to amphibian osmolality. The cells were incubated at 27 °C with 5% CO_2_ in the presence of respective stimuli.

### 2.4. Pathogen-Associated Molecular Pattern (PAMP) Stimulation and FV3 Infectoins of X. laevis Tadpole and Adult Frog Skin and Kidney Cells

Isolated *X. laevis* tadpole (*N* = 5) and adult frog (*N* = 5) skin and kidney cells were seeded at densities of 10^5^ cells/well into 96 well plates and were stimulated with the following PAMPs. There was a high molecular weight of Poly I:C (RIG-I/MDA-5 agonist, 10 μg/mL); FSL-1 lipoprotein (toll-like receptor, TLR2/6 agonist, 1 μg/mL); LPS from *E. coli* K12 (TLR4 agonist, 10 μg/mL); Imiquimod (TLR7 agonist, 5 μg/mL); ssRNA 40 (TLR8 agonist, 5 μg/mL); and CpG ODN 2006 (TLR9 agonist, 5 μg/mL). The cells were also stimulated with 10 μg/mL of the following cytosolic DNA sensor agonists: HSV-60 (60 bp oligonucleotide containing viral DNA motifs), VACV-70 (70 bp oligonucleotides containing viral DNA motifs), and IFN-stimulatory DNA (ISD, 45-bp non-CpG oligomer from the *Listeria monocytogenes* genome). The Poly I:C, HSV-60, VACV-70, and ISD were added to the cells in conjunction with the LyoVec transfection reagent. All of the treatments and stimulation conditions were performed in accordance to the manufacturer’s instructions (InvivoGen, San Diego, CA, USA). The tadpole and adult frog skin and kidney cells were incubated with the respective PAMPs for 6 h before they were harvested and processed for RNA isolation and cDNA synthesis.

The isolated *X. laevis* tadpole (*N* = 5) and adult frog (*N* = 5) skin and kidney cells were seeded at densities of 10^5^ cells/well into 96 well plates and mock-infected (saline) or infected at a multiplicity of infection (MOI) of 0.5 plaque forming units (PFU) of FV3 per cell for 6 h. Subsequently, the cells were washed in saline, collected by centrifugation and processed for RNA and DNA isolation.

### 2.5. Isolation of RNA and DNA Form Tadpole and Adult Frog Tissues and Cells

For all experiments, tadpole and adult tissues and cells were placed in Trizol reagent (Invitrogen, Carlsbad, CA, USA), flash frozen on dry ice and stored at −80 °C until RNA and DNA isolation. The tissues and cells were homogenized by passage through progressively higher gage needles, and RNA isolation was performed using the Trizol (Invitrogen), according to manufacturers’ directions. Following the RNA extraction, the remaining Trizol layer was mixed with a back extraction buffer (4 M guanidine thiocyanate, 50 mM sodium citrate, 1 M Tris pH 8.0) and centrifuged to isolate the DNA containing aqueous phase. The DNA was precipitated overnight with isopropanol, pelleted by centrifugation, washed with 70% ethanol, and resuspended in TE (10 mM Tris pH 8.0, 1 mM EDTA) buffer. The DNA was then purified by phenol-chloroform extraction, resuspended in molecular grade water and quantified.

### 2.6. Quantitative Gene Expression Analyses

The total RNA and DNA were extracted from the frog tissues and cells using the Trizol reagent and following the manufacturer’s directions (Invitrogen), and all of the cDNA syntheses were performed using iScript cDNA synthesis kits, in accordance to manufacturers’ directions (Bio-Rad, Hercules, CA, USA), using 500 ng of the total RNA. The gene expression analyses of type I and type III *IFNs*, pattern recognition receptors (*PRRs*) and FV3 immediate early (82R), delayed-early (95R), and late (93L) genes was performed using the delta^delta CT method, with all of the gene expressions examined relative to the *GAPDH* endogenous control. The expression of the individual *IFN* genes was normalized against the respective control expression.

The FV3 viral loads were determined by the absolute qPCR against the DNA (isolated from infected animals), using a serially diluted standard curve. Briefly, the pGEM-T vector (Promega, Madison, WI, USA) bearing the FV3 vDNA Pol (ORF 60R) fragment was serially diluted to yield 10^1^–10^8^ vDNA Pol fragment-containing plasmid copies. These were used as the standard curve in the subsequent absolute qPCR assays of the FV3 genomic DNA copies, using primers against the FV3 DNA polymerase. All of the absolute qPCR analyses were performed using 50 ng of total isolated DNA.

All of the experiments were performed using the CFX96 Real-Time System and iTaq Universal SYBR Green Supermix. The BioRad CFX Manager software (SDS) was used for all of the expression analyses. All of the primers were validated prior to use.

### 2.7. Statistical Analysis

The statistical analysis was performed using a one-way analysis of variance (ANOVA) and post hoc *t*-test, using Vassar Stat (http://faculty.vassar.edu/lowry//anova1u.html) and Graph Pad (https://www.graphpad.com/quickcalcs/ttest1.cfm) statistical programs, respectively. A probability level of *p* < 0.05 was considered significant.

## 3. Results

### 3.1. Tadpoles and Adult Frogs Exhibit Distinct Skin Cell, But Not Kidney Cell PRR Gene Expression

**Figure 1:**
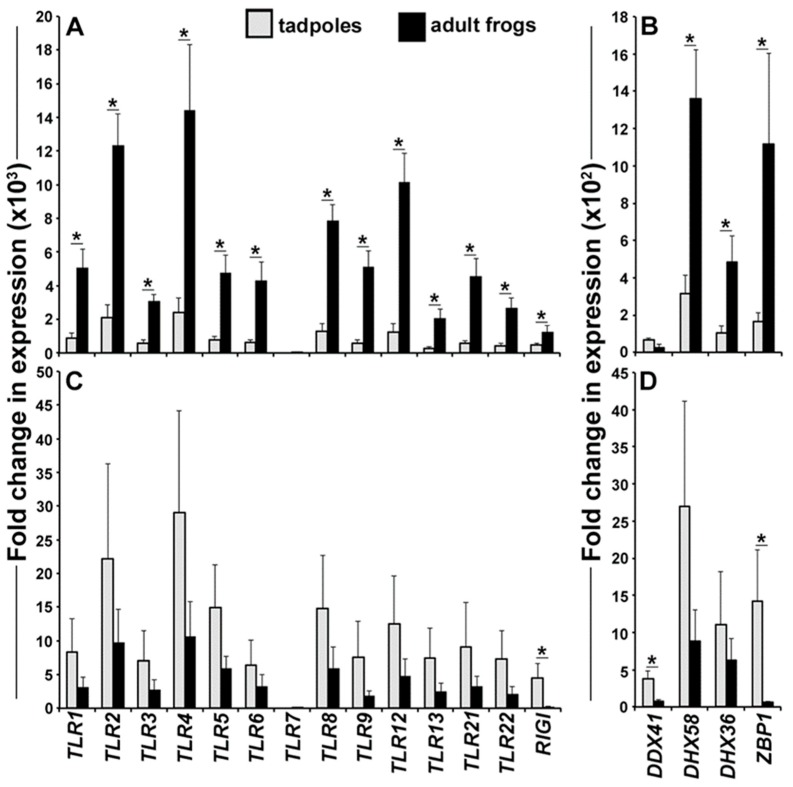
Pathogen pattern recognition receptor (PRR) expression in tadpole and adult frog skin and kidney cells. Tadpoles (Nieuwkerk Faber, NF 54, *N* = 5) and adult frog (1 year old, *N* = 5) skin and kidney cells were examined for their gene expression of a panel of pathogen pattern recognition receptors. The expression analyses included (**A**) skin cell *TLR*, *RIGI*, and (**B**) *cytosolic DNA sensor (CDS)*, genes as well as (**C**) kidney *TLR*, *RIGI*, and (**D**) CDS mRNA levels. All of the gene expressions were compared to the *GAPDH* control and normalized against the lowest expressed *PRR* (*TLR7*) within the respective cells. All of the results are presented as means + SEM. Significant differences between the respective experimental groups are denoted by overhead lines and asterisks (*). *p* < 0.05.

We previously showed that *X. laevis* tadpoles and adult frogs undergo distinct type I and type III *IFN* responses at their skin following water exposure to FV3 [19]. Typically, the *IFN* responses are mounted following the recognition of viral (and other types of pathogen) infections by pathogen pattern recognition receptors (PRRs) [5,22,23,24]. To examine the capacities of tadpole and adult frog skin cells to recognize viral (as well as other) infections, we isolated the respective cells and examined their gene expression of a panel of *PRR* genes, including those responsible for the recognition of viral as well other types of pathogens (Figure 1). With the exception of the *TLR7*, adult *X. laevis* possessed a significantly greater gene expression of mammalian-like (*TLR1–9*) and frog-specific (*TLR12*, *13*, *21*, *22*) TLRs as well as the retinoic acid-inducible gene I (*RIGI*, Figure 1A). Moreover and compared to tadpoles, the adult frogs exhibited significantly greater skin mRNA levels of three (*DHX58, DHX36, ZBP1*) of the four examined cytosolic DNA sensor genes (Figure 1B).

The amphibian kidney is a principal viral target during FV3 infections of both tadpoles and adult frogs [21] and our recent work indicates that FV3 disseminates to tadpole and adult kidneys soon after water infection [19]. It is thus important to gain an understanding of the tadpole and adult frog antiviral responses within this tissue. Accordingly, we isolated the tadpole and adult frog kidney cells and examined their expression of the *PRR* genes (Figure 1C,D). In contrast to the tadpole and adult *X. laevis* skin *PRR* expression, the tadpoles and adults did not significantly differ in the majority of their kidney cell-expressed *PRRs* (Figure 1C,D). Notable exceptions of this were the *RIGI* (Figure 1C), *DDX41*, and *ZBP1* (Figure 1D) transcripts, which were significantly more expressed in tadpole kidneys compared to those of the adult frogs.

### 3.2. Tadpoles and Adult Frog Skin and Kidney Cells Mount Distinct IFN Responses to PAMP Stimulation

**Figure 2:**
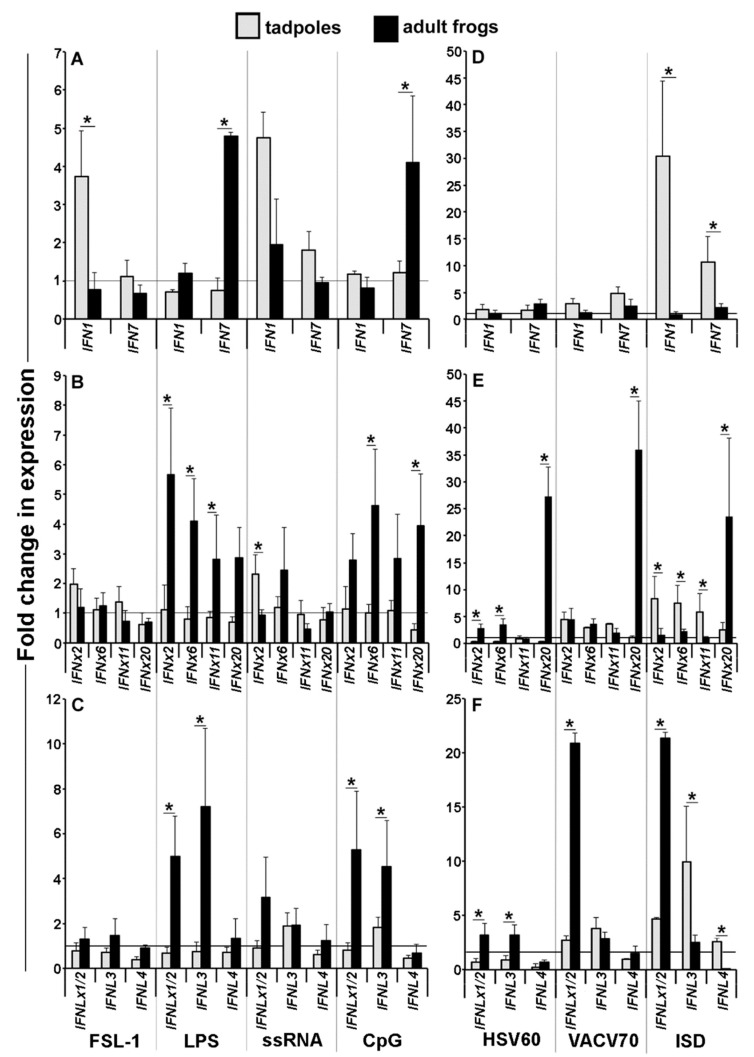
Tadpoles and adult frog skin cells mount distinct interferon *(IFN)* responses to PAMP stimulations. Tadpoles (NF 54, *N* = 5) and adult frog (1 year old, *N* = 5) skin cells were isolated and stimulated with a panel of pathogen-associated molecular patterns (PAMPs) for 6 h, and the IFN gene expression was examined. (**A**) *IFN1* and *IFN7*; (**B**) *IFNx2*, *IFNx6*, *IFNx11*, *IFNx20* (**C**) *IFNLx1/2*, *IFNL3*, and *IFNL4* gene expression, following stimulation with TLR and RIGI-specific PAMPs. (**D**) *IFN1* and *IFN7*; (**E**) *IFNx2*, *IFNx6*, *IFNx11*, and *IFNx20*; (**F**) *IFNLx1/2*, *IFNL3*, and *IFNL4* gene expression, following stimulation with CDS agonists. All of the gene expressions were compared to the *GAPDH* control, normalized against the mock-infected expression of the respective genes within given tissues. All of the results are presented as means + SEM. Significant differences between the respective experimental groups are denoted by overhead lines and asterisks (*). *p* < 0.05.

**Figure 3:**
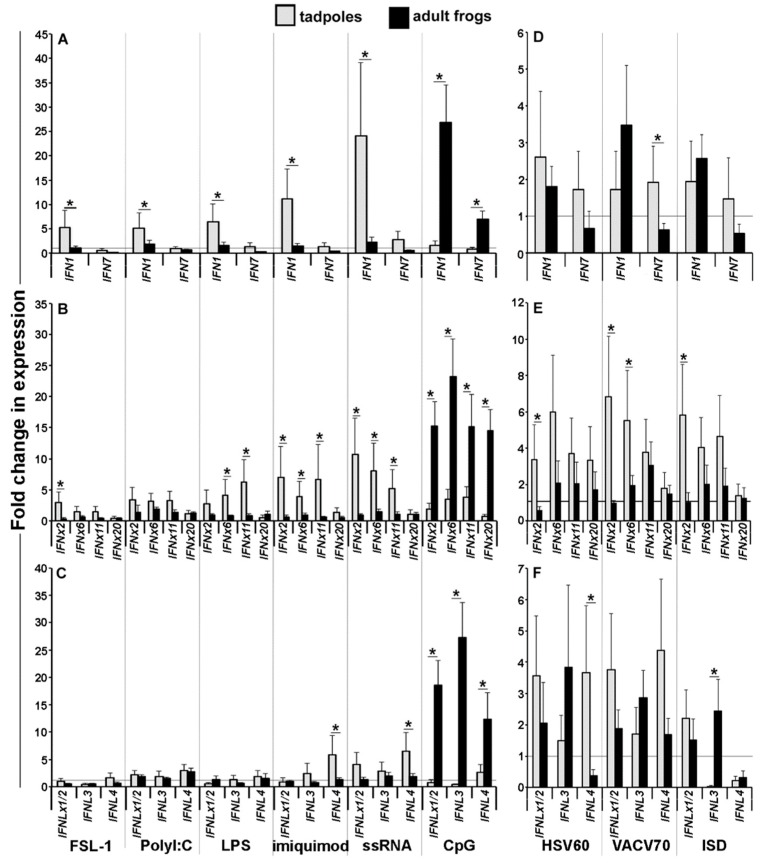
Tadpoles and adult frog kidney cells mount distinct *IFN* responses to PAMP stimulations. Tadpoles (NF 54, *N* = 5) and adult frog (1 year old, *N* = 5) skin cells were isolated and stimulated with a panel of PAMPS for 6 h, and the IFN gene expression was examined. (**A**) *IFN1* and *IFN7*; (**B**) *IFNx2*, *IFNx6*, *IFNx11*, and *IFNx20*; (**C**) *IFNLx1/2*, *IFNL3*, and *IFNL4* gene expression following stimulation with TLR and RIGI-specific PAMPs. (**D**) *IFN1* and *IFN7*; (**E**) *IFNx2*, *IFNx6*, *IFNx11*, and *IFNx20*; (**F**) *IFNLx1/2*, *IFNL3*, and *IFNL4* gene expression following stimulation with CDS agonists. All of the gene expressions were compared to the *GAPDH* control, normalized against the mock-infected expression of the respective genes within the given tissues. All of the results are presented as means + SEM. Significant differences between the respective experimental groups are denoted by overhead lines and asterisks (*). *p* < 0.05.

The *Xenopus* frogs were recently shown to encode broad repertoires of intron-containing and intronless type I and type III *IFNs* (*IFN IFNx*, *IFNL*, and *IFNLx*, respectively: [13]). Considering the differences in the tadpole and adult frog skin, but not their kidney cell expression of the *PRRs* (Figure 1A,B), we next examined the expression of representative *X. laevis* type I and III *IFN* genes from distinct phylogenetic clades (Figure S1) in the isolated tadpole and adult frog skin and kidney cells that were stimulated with viral and non-viral pathogen-associated molecular patterns (PAMPs; Figure 2 and Figure 3). We chose primarily viral PAMPs (Poly I:C, imiquimod, ssRNA, HSV-60, VACV-70, ISD), but also included several bacterial PAMPs (FSL-1, LPS, CpG), as some the amphibian IFNs are though to mediate antibacterial roles [13].

The PAMP-stimulated tadpoles and adult frog skin cells exhibited the upregulation of distinct *IFN* genes (Figure 2). For example, the tadpole, but not the adult skin cells stimulated with the FSL-1 lipoprotein upregulated their *IFN1* gene expression (Figure 2A). Conversely, the adult frog skin cells were much more responsive to LPS than the tadpole skin cells, as the former upregulated the *IFN7*, *IFNx2*, *IFNx6*, *IFNx11*, *IFNx20*, *IFNLx1/2*, and *IFNL3* in response to this stimulus (Figure 2A–C). The tadpole skin cells stimulated with ssRNA exhibit greater expression increases of *IFN1* and *IFNx2* genes than adult frog skin cells (Figure 2A,B). The adult frog skin cells showed greater transcriptional increases in the *IFN7*, *IFNx6 IFNx20*, *IFNLx1/2*, and *IFNL3* gene expression (Figure 2A–C).

Compared to the adult frog skin cells, the tadpole skin cells possessed much more robust gene expression increases in *IFN1*, *IFN7*, *IFNx2 IFNx6 IFNx11 IFNLx1/2*, and *IFNL3* (Figure 2D–F) in response to the ISD cytosolic DNA sensor (CDS) agonist. By contrast, the adult frog skin cells exhibited greater gene expression increases in *IFNx2*, *IFNx6*, *IFNx20*, *IFNLx1/2*, and *IFNL3* in response to the HSV60 DNA sensor agonist; more upregulated expression of *IFNx20* and *IFNLx1/2* in response to the VACV-70 DNA sensor agonist and a further increased *IFNx20* and *IFNLx1/2* in response to ISD (Figure 2E,F).

The tadpole and adult frog skin cells did not exhibit significant changes in any of the examined *IFN* genes in response to Poly I: C or imiquimod stimulation.

The tadpole kidney cells were much more responsive to several PAMPs than the adult kidney cells (Figure 3). For example, the tadpole kidney cells exhibited greater expression increases in *IFN1* and *IFNx2* in response to FSL-1; greater expression of IFN1 in response to Poly I:C; more robust *IFN1*, *IFNx6*, *IFNx11*, and *IFNL4* in response to LPS and greater transcriptional increases in *IFN1*, *IFNx2*, *IFNx6*, *IFNx11*, and *IFNL4* in response to imiquimod. The tadpole kidney cells also showed further elevated *IFN1*, *IFNx2*, *IFNx6*, *IFN11*, and *IFNL4* gene expression in response to ssRNA (Figure 3A–C). In contrast, the adult frog kidney cells responded significantly more robustly to the CpG stimulation by upregulating greater mRNA levels of all of the examined *IFN* genes (Figure 3A–C).

The tadpole and adult frog kidney cell responses to the DNA sensor agonists were more variable and showed fewer differences (Figure 3D–F). Compared with the adult cells, tadpole kidney cells showed significantly greater increases in *IFNx2* and *IFNL4* in response to HSV60; greater *IFN7*, *IFNx2*, and *IFNx6* responses to VACV-70 and a more robust *IFNx2* expression in response to ISD. By contrast, the adult frog kidney cells only possessed greater increases in *IFNL3* in response to ISD (Figure 3F).

### 3.3. Tadpoles and Adult Frog Skin and Kidney Cells Mount Distinct IFN Responses to FV3

**Figure 4:**
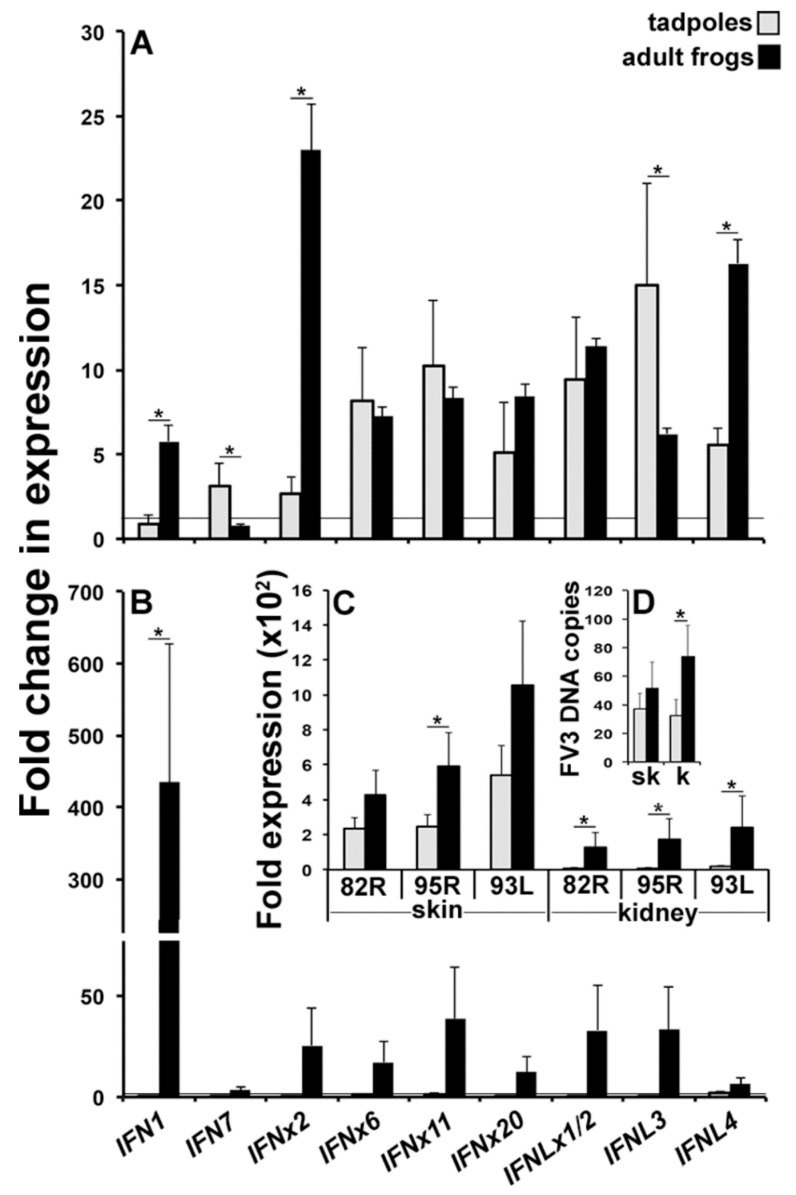
Tadpoles and adult frog kidney cells mount distinct *IFN* responses following FV3 infections. Tadpoles (NF 54, *N* = 5) and adult frog (1 year old, *N* = 5) skin cells were isolated and infected with FV3 (0.5 MOI) for 6 h, and were examined for skin (**A**) and (**B**) kidney cell expressions of *IFN* genes as well as (**C**) skin and kidney cell expressions of FV3 genes and their (**D**) FV3 DNA loads. All of the gene expressions were compared to the *GAPDH* control, normalized against the mock-infected expression of the respective genes within given tissues. All of the results are presented as means + SEM. Significant differences between the respective experimental groups are denoted by overhead lines and asterisks (*). *p* < 0.05.

In light of the very distinct *IFN* responses mounted by the tadpole and adult frog skin and kidney cells to distinct PAMPs, we next examined the *IFN* gene expression of these respective cells following FV3 infection. Accordingly, we isolated the tadpole and adult frog skin and kidney cells, infected them for 6 h with FV3 (0.5 MOI, relatively low infection) and examined their type I and III *IFN* gene expression (Figure 4).

While the FV3-infected tadpole skin cells exhibited significantly more upregulated gene expression of *IFN7* and *IFNL3*, the virus-challenged adult frog skin cells possessed much greater increases in their *IFN1*, *IFNx2*, and *IFNL4* transcripts (Figure 4A). The FV3-infected adult frog kidney cells exhibited a greater expression of all of the examined *IFN* genes, albeit only significantly so for *IFN1* (Figure 4B).

In order to confirm that the isolated skin and kidney cells indeed supported the FV3 replication, we examined the respective FV3-infected tadpole and adult frog cells for the expression of the FV3 immediate early (82R), delayed-early (95R), and late (93L) genes as well as their FV3 DNA loads (Figure 4C,D). Akin to our previous observations in animal tissues [19], the adult frog skin and kidney cells supported a greater FV3 gene expression (significantly so for skin 95R and kidney 82R, 95R, and 93L) than the respective tadpole cells (Figure 4C). Moreover, while the tadpole and adult frog skin cells did not differ their FV3 DNA loads, the adult frog kidney cells exhibited greater viral loads than the tadpole kidney cells (Figure 4D).

### 3.4. Tadpoles and Adult Frog Skin and Kidney Tissues Differ in Their IFN Responses to FV3

**Figure 5:**
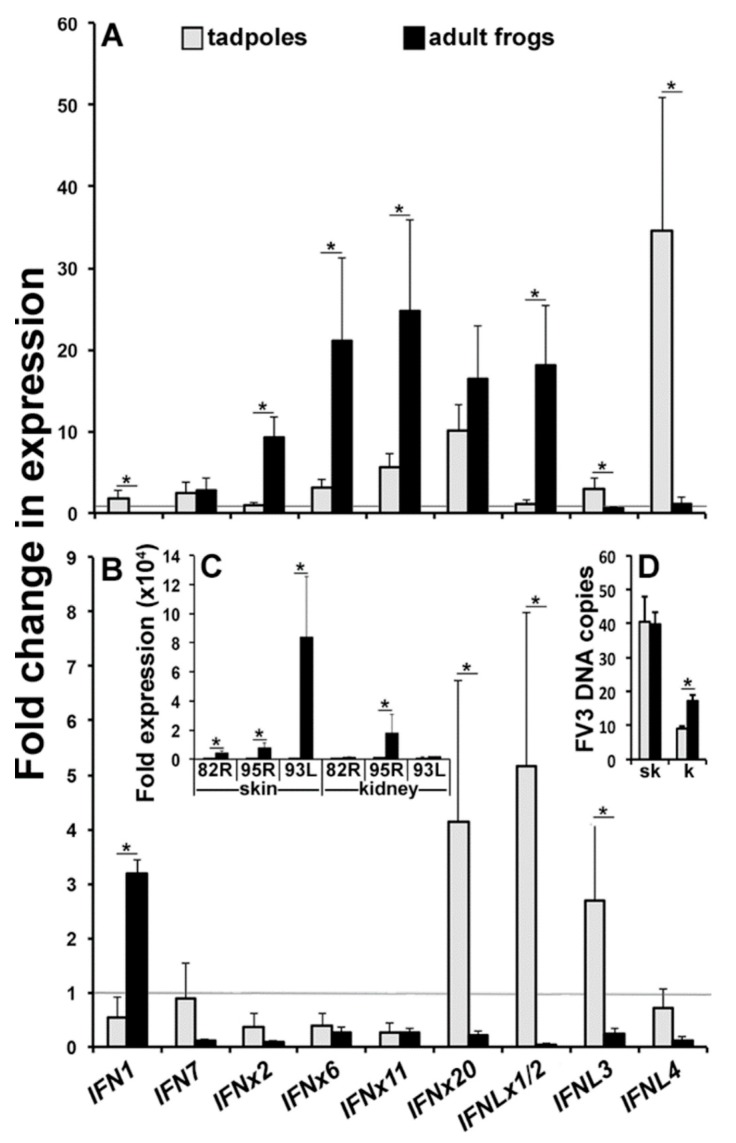
Tadpoles and adult *X. laevis* mount distinct *IFN* responses to FV3. Tadpoles (Nieuwkoop and Faber, NF stage 54, *N* = 5) and adult frogs (1 year old, *N* = 5) were challenged by water bath with 10^6^ PFU of FV3 per ml of water or mock infected (water), and after 6 h of infection, their (**A**) skin and (**B**) kidney tissues were examined for the *IFN* gene expression, (**C**) FV3 gene expression, and (**D**) FV3 DNA viral loads. All of the gene expressions were compared to the *GAPDH* control, normalized against the mock-infected expression of the respective genes within given tissues. All of the results are presented as means + SEM. Significant differences between the respective experimental groups are denoted by overhead lines and asterisks (*). *p* < 0.05.

The above studies utilized isolated animal cells and therefore recapitulated the *IFN* responses by those tadpole and adult skin and kidney cells that would be present within the respective tissues at a steady state. However, the above expression studies cannot account for the potential additional *IFN*-expressing cell population that may be recruited into these respective tadpole and adult tissues upon the FV3 challenge. To reconcile this possibility, we infected tadpoles and adult frogs with FV3 by water bath and 6 h later examined the gene expression of type I and type III *IFNs* in their skin and kidney tissues (Figure 5A,B).

The tadpoles and adult frogs mounted drastically distinct *IFN* responses in their skin tissues following this immune challenge, with the tadpoles exhibiting a significantly greater upregulation of the *IFN1*, *IFNL3*, and *IFNL4* gene expression and the adult frogs possessing significantly greater increases in the *IFNx2*, *IFNx6*, *IFNx11*, and *IFNLx1/2* transcripts (Figure 5A). 

While the gene expression patterns of *IFNx2* and *IFNL4* were similar between the FV3-challeneged tadpole and adult frog (isolated) skin cells and skin tissues, it was quiet striking that all of the other examined *IFNs* possessed very different expression patterns between the cells and tissues (Figure 4A and Figure 5A).

The tadpole and adult frog kidney *IFN* responses were markedly different from those seen at their respective skin tissues (Figure 5A,B). While the tadpoles exhibited significantly greater changes in their expression of *IFNx20*, *IFNLx1/2*, and *IFNL3*, the adult frogs mounted exclusively an *IFN1* response at this site (Figure 5B).

Akin to the FV3-challenegd tadpole and adult frog (isolated) kidney cells, the FV3-infected adults exhibited greater gene expression of *IFN1* than seen in the virally challenged tadpole kidneys (Figure 4B and Figure 5B). Intriguingly and as observed with the respective skin cells and skin tissues, the FV3-infected tadpoles and adult frogs possessed very different kidney expression patterns of all of the other examined *IFN* genes, as compared to the respective isolated and virus-infected cell populations (Figure 4B and Figure 5B).

Consistent with our past studies [19] and in corroboration with our in vitro skin and kidney cell infection studies (Figure 4); tadpoles possessed markedly lower skin and kidney tissue expression of FV3 genes and significantly lower kidney but not skin FV3 loads (Figure 5C,D), possibly owing to their unique kidney *IFN* responses. Considering that the FV3 replication time is around 12–24 h, we predict that the detected viral loads represent FV3 that has disseminated to the animal kidney from the skin, gills, and/or gastrointestinal tissues; all of which would be in contact with, and potential entry points for FV3.

## 4. Discussion

It is interesting to consider that of all of the vertebrates examined to date (many of which are now known to be infected by ranaviruses), the *Xenopudinae* frogs possess the most diverse and expanded repertoires of type I and type III IFN cytokines [12,13]. It is perhaps more interesting to speculate why these animals have diverged to encode so many distinct IFNs, and in turn, what the functional roles of these respective moieties may be. Notably, the ranavirus pathogens are significant contributors to the worldwide amphibian declines [1,2,3], while the anuran (frogs and toads) tadpoles are more susceptible to these infections than the adult frogs of the respective species [4,5,6,7,8]. Thus, gaining greater insights into the potential roles of these expanded *IFN* repertoires during amphibian tadpole and adult frog antiviral responses are imperative. Indeed, the results presented here indicate that the *X. laevis* tadpoles and adults mount vastly different *IFN* responses to FV3, as well as to distinct viral PAMPs; possibly accounting for the susceptibility differences between these developmental stages.

It is notable that following FV3 challenge, the tadpoles responded predominantly with intron-containing type III *IFNs* at their skin, whereas the adult frogs mounted more robust intronless type I and type III *IFN* responses. Similar results were derived when the adult skin cells were stimulated with the cytosolic DNA sensor agonists (FV3 is a DNA virus), suggesting that the adult frogs utilize these particular IFNs when dealing with DNA viruses. Conversely, the FV3-infected tadpole kidneys possessed significantly more upregulated levels of an intronless type I *IFNs*, as well as an intronless and an intron-containing type III *IFNs*, whereas the FV3-infected adult frogs mounted an exclusively intron-containing type I *IFN* response within their kidneys. Both of the amphibian skin and kidney tissues undergo drastic changes during metamorphosis, including changes in their cellular composition [25,26]. Presumably, these differences are reflected in the distinct tadpole and adult frog *IFN* responses and may mark differences in the ability of the two developmental stages to control the FV3. For example, after 6 h of infection, the tadpoles possessed lower kidney FV3 loads than the adult frogs, which may be accounted for by their distinct *IFN* responses at this site. Indeed, the tadpole kidney cells responded more robustly to the viral PAMPs with their *IFN* gene expression than the kidney cells from adult frogs, possibly indicating that the *X. laevis* tadpoles are more effective at recognizing and containing viral pathogens at this site. It is notable that the adult frog kidney cells possessed significantly greater IFN responses to CpG, suggesting that the adult kidney may be more equipped at dealing with bacterial infections. Many of the *X. laevis IFNs* are thought to participate in antibacterial responses [13] and it would seem that the adult frogs heavily rely on some of these *IFNs* during bacterial infections.

Our expression analyses indicate that at the skin, the adult frogs express greater levels of most of the examined PRRs. While this does not explain the drastic differences in the tadpole and adult *IFN* responses, it may indicate the presence of distinct cellular populations. Notably, less PRR expression differences were observed between the tadpole and adult kidney tissues. This in turn suggests that the distinct tadpole and adult *IFN* responses may be not only a function of the cellular composition of the respective tissues, but may reflect the distinct immune strategies of the two development stages. Unfortunately, at present, the cellular compositions of the tadpole and adult frog skin and kidney tissues remain poorly defined. Thus, further research into this aspect of amphibian physiology will help define the facets of pathogen susceptibility and may explain the differences in the *IFN* responses reported here.

The tadpole and adult frog skin cells did not respond to Poly I:C or imiquimod stimulation, despite expressing the putative *PRRs* that recognize these agonists (RIG-I/TLR3 and TLR7, respectively), whereas the tadpole and adult kidney cells responded to these respective stimuli. Interestingly, the stimulation of tadpole and adult skin cells with another TLR7 agonist, ssRNA also resulted in relatively modest *IFN* responses, with fewer *IFN* genes being upregulated than observed with the other PRR agonists. Possibly, these results reflect the transfection efficiencies of these compounds into the tadpole and adult frog skin (but not kidney) cells. Alternatively, as all of these agonists mimic the RNA viruses/viral products, together our results may suggest that the *X. laevis* are less sensitive or less effective at recognizing viral RNAs. While *RIGI* and *TLR3* (PRRs that recognize dsRNA/Poly I:C) were expressed in animal skin tissues, the putative imiquimod/ssRNA receptor, *TLR7* was very poorly expressed in both the tadpole and adult skin tissues. This would be a convenient explanation for the observed non-responsiveness to imiquimod, except that the animal kidneys, despite possessing a meager *TLR7* expression, exhibited robust *IFN* responses to imiquimod and ssRNA. Considering that fresh water environments such as those inhabited by amphibians contain vast amounts of biologically diverse RNA viruses [27], the non-responsiveness of these animals to viral RNA mimics may possibly reflect their desensitization to RNA viruses. This would be an intuitive way for an organism that is constantly in contact with RNA viruses to prevent being in a continual state of immune activation. Indeed, our observations indicate that the tadpole and adult kidney cells readily respond to the viral RNA mimics. Thus, while it is also possible that distinct results may be obtained with different concentrations of these PAMPs or when examining other antiviral genes downstream of the PRR activation, we propose that this sensitization may at least in part be responsible for the differences in the *X. laevis* skin and kidney responses. It would be most interesting to see whether the fish skin cells are likewise desensitized to the RNA viruses.

The notion of sensitization to environmental stimuli may also be extended to tadpole and adult skin responsiveness to LPS. Aquatic vertebrates are notorious for their resistance to LPS stimulation and much larger concentrations of this gram-negative bacterial cell wall component are required to activate immune cells of aquatic species [28]. Interestingly, the tadpole skin cells were completely unresponsive to LPS whereas the adult frog skin cells mounted robust *IFN* responses to LPS. Presumably, since the tadpole skin is a much weaker barrier than the skin of the adult frogs combined with the fact that these animals reside in bacteria-rich environments may account for why tadpoles may be desensitized against immune recognition/activation in response to LPS.

The comparison of the *IFN* gene expression in the isolated tadpole and adult frog cells and the infected animal tissues indicate that while the respective resident cells (the identity of which remains awaits the development of new *X. laevis*-specific reagents) are capable of recognizing FV3 and other pathogen threats and mounting specific *IFN* responses, both tadpoles and adults rely on the recruitment of additional cell populations to coordinate their *IFN* responses. Indeed, while the responses of some *IFNs* (skin *IFNx2* and *IFNL4*; kidney *IFN1*) appear to be mediated by the cells that are present within the skin and kidney tissues of healthy animals, the drastic differences reported here between the FV3-challeneged cells and the tissues isolated from infected animals suggest that the additional *IFN*-expressing populations are rapidly (within 6 h) recruited to the infection sites to mediate the *IFN* responses. It will be most invaluable to learn which tadpole and adult frog immune (and possibly non-immune) cell types are recruited to the sites of FV3 infection and what the roles of the respective *IFNs* produced by the resident and recruited cell types during anti-ranavirus immune responses are.

As we move forward to gain greater insights into the infection, replication, and immune evasion strategies of FV3 and other ranaviruses, it is imperative that we continually re-evaluate our understanding of these pathogens and acknowledge the fact that there is much that we do not know about how these pathogens replicate in the different cells and tissues of their numerous cold-blooded vertebrate hosts. In so doing, it also important to not build false dogmas upon previous literature, some of which may have employed much less-sensitive approaches (than presently available) in the context of cell lines that may not accurately reflect the natural cell tropisms of these pathogens, or representing one alternative cellular host to these viruses. Indeed, it has been more recently demonstrated that the modes of FV3 replication and the ensuing cell cytotoxicity (including apoptosis) and immune responses greatly vary with the infected cell type [29]. Likewise, here we show that the FV3 replication and the induction of antiviral immune responses are highly variable, depending on the host developmental stage and the cell types examined. Considering the above, combined with the promiscuity with which ranaviruses infect many distinct cell types within the drastically different organisms; we beseech researchers studying these pathogens to remain flexible in their perspectives of the ranavirus cell tropisms, replication strategies and resulting cytotoxicities.

From an evolutionary standpoint, it is intriguing to consider that amphibians have diverged to encode so many distinct type I and type III *IFNs.* While the evolutionary reason for this stark divergence from other vertebrates is difficult to speculate upon, clearly some aspect of their physiology and immune pressures has been selected for the expansion and maintenance of these diverse gene families. When considering that the tadpoles and adult frogs have drastically different physiologies and pathogen susceptibilities, it is intuitive that they would take advantage of the distinct repertoires of these *IFN* cytokine in response to unique immune challenges. In turn, gaining a greater insight into the functional significance of those *IFNs* that are respectively utilized by tadpoles and adult frogs will yield new insights into the tadpole and adult immune efficacies and to the evolution of these cytokine families.

## Supplementary Materials

The following are available online at http://www.mdpi.com/1999-4915/10/7/372/s1, Figure S1: The phylogenetic tree was constructed using the neighbor joining method and bootstrapped 10,000 times (denoted as %s). The X. laevis IFNs chosen for gene expression analyses are indicated by arrows.
