# Supplementary material for: Amphibian (Xenopus laevis) Tadpoles and Adult Frogs Differ in Their Use of Expanded Repertoires of Type I and Type III Interferon Cytokines
Source: Viruses. 2018 Jul 17;10(7):372. doi: 10.3390/v10070372 (PMC6070924; doi:10.3390/v10070372)
Supplement: Supplementary file 1 [file viruses-10-00372-s001.pdf]

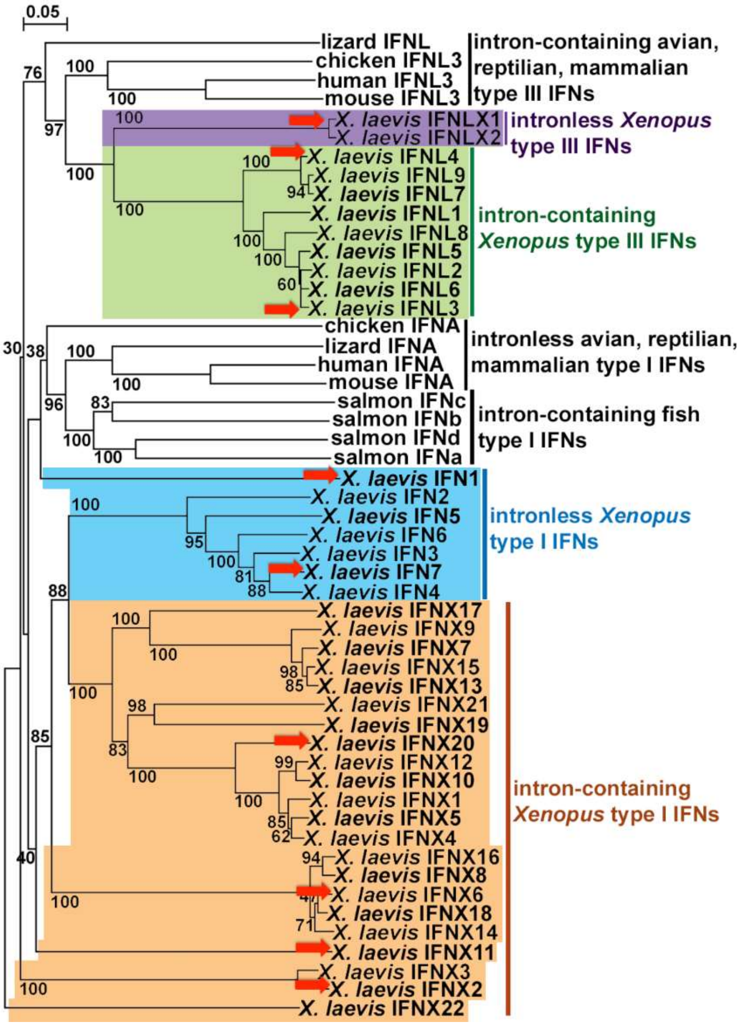

Supplemental Figure S1. The phylogenetic tree was constructed using the neighbor joining method and bootstrapped 10,000 times (denoted as %s). The *X. laevis* IFNs chosen for gene expression analyses are indicated by arrows.
